# Long-term Intake of Pasta Containing Barley (1–3)Beta-D-Glucan Increases Neovascularization-mediated Cardioprotection through Endothelial Upregulation of Vascular Endothelial Growth Factor and Parkin

**DOI:** 10.1038/s41598-017-13949-1

**Published:** 2017-10-18

**Authors:** Valentina Casieri, Marco Matteucci, Claudia Cavallini, Milena Torti, Michele Torelli, Vincenzo Lionetti

**Affiliations:** 1 Institute of Life Sciences, Scuola Superiore Sant’Anna, Pisa, Italy; 2 ATTRE (Advanced Therapies and Tissue Regeneration) Laboratory, Innovation Accelerator CNR, Bologna, Italy; 3 Research and Development Unit, Pastificio Attilio Matromauro Granoro s.r.l, Corato, Italy; 4 UOS Anesthesia and Intensive Care, Fondazione Toscana “G. Monasterio”, Pisa, Italy

## Abstract

Barley (1–3)β-D-Glucan (BBG) enhances angiogenesis. Since pasta is very effective in providing a BBG-enriched diet, we hypothesized that the intake of pasta containing 3% BBG (P-BBG) induces neovascularization-mediated cardioprotection. Healthy adult male C57BL/6 mice fed P-BBG (n = 15) or wheat pasta (Control, n = 15) for five-weeks showed normal glucose tolerance and cardiac function. With a food intake similar to the Control, P-BBG mice showed a 109% survival rate (P < 0.01 vs. Control) after cardiac ischemia (30 min)/reperfusion (60 min) injury. Left ventricular (LV) anion superoxide production and infarct size in P-BBG mice were reduced by 62 and 35% (P < 0.0001 vs. Control), respectively. The capillary and arteriolar density of P-BBG hearts were respectively increased by 12 and 18% (P < 0.05 vs. Control). Compared to the Control group, the VEGF expression in P-BBG hearts was increased by 87.7% (P < 0.05); while, the p53 and Parkin expression was significantly increased by 125% and cleaved caspase-3 levels were reduced by 33% in P-BBG mice. *In vitro*, BBG was required to induce VEGF, p53 and Parkin expression in human umbelical vascular endothelial cells. Moreover, the BBG-induced Parkin expression was not affected by pifithrin-α (10 uM/7days), a p53 inhibitor. In conclusion, long-term dietary supplementation with P-BBG confers post-ischemic cardioprotection through endothelial upregulation of VEGF and Parkin.

## Introduction

Early primary coronary revascularization significantly restores the blood supply to the ischemic myocardium and limits the loss of cardiac cells^1^, yet its clinical efficacy varies widely among patients^2^ and with increasing age^3^. An individual’s tolerance to the post-ischemic reperfusion insult of the heart depends on the variation in density of the native coronary collaterals^4,5^. These collaterals lead to retrograde perfusion of the obstructed coronary tree by limiting the magnitude of the myocardial ischemia/reperfusion (I/R) injury in animal models^6^ and also in humans^7^. The rarefaction of the native coronary collaterals limits the efficacy of coronary reperfusion^8^ and may worsen the outcome of ischemic patients who are not eligible for reperfusion therapy^5,9^.

De novo formation of coronary collaterals thus has the potential to prevent myocardial injury at early stages of post-ischemic reperfusion^10^. The formation of collateral vessels is attributable to the production of the vascular endothelial growth factor (VEGF)^11^, which plays a key role in improving angiogenic sprout formation^12^ and endothelial cell/cardiomyocyte cross-talk^13^. In addition, autocrine endothelial VEGF is essential for normal metabolic functions and endothelial cell preservation^14^. Importantly, the regulation of endothelium-cardiomyocyte cross-talk may prevent the post-ischemic decay of cardiomyocyte contractility^15^, even in a VEGF- dependent manner^16^. In fact, the paracrine actions of VEGF promptly affect the function of adjacent cardiac cells with a high potential for myocardial repair^17^.

Effective de novo formation of coronary collaterals is dependent on endothelial cell integrity, which is maintained by Parkin, an E3-ligase that combines target proteins with ubiquitin, leading to their proteasomal degradation, and protects mitochondrial function^18^. Parkin deficiency abolishes the ischemic preconditioning-induced cardioprotection^19^ and it reduces cardiac tolerance to hypoxia^20^. Although gene therapy increases the myocardial expression of VEGF^21^ or Parkin^20^ by protecting the heart from ischemic insult, non-invasive approaches to upregulating these proteins remain a desirable goal. Recently, we demonstrated that long-term exposure of human endothelial cells to 3% BBG increases manganese superoxide dismutase (MnSOD) expression and nitric oxide (NO) production, which promote the formation of new vessels^22^. Although a previous *in vitro* study revealed that VEGF induces endothelial MnSOD expression^23^, it is still unknown whether sustained dietary intake of BBG promotes cardioprotection *in vivo* through endothelial upregulation of VEGF and Parkin.

Short-term oral intake of a higher dose of β-1.3/1.6-Glucan isolated from Saccharomyces cerevisae prevents cardiac injury in pigs^24^ and in humans^25^ subject to myocardial I/R. The cardioprotective effects of long-term dietary intake of water-soluble barley-derived (1.3) β-D-Glucan (BBG), whose antioxidant activity is higher than fungal β-Glucan^26^, have not yet been investigated.

Since pasta plays a key role in human nutrition and is very effective in providing a BBG-enriched diet^27^, we tested whether the daily intake of diet supplemented with pasta containing 3% BBG (P-BBG) noninvasively increases coronary collaterals and cell survival through the simultaneous endothelial expression of VEGF and Parkin, which might mitigate the cardiac injury due to post-ischemic reperfusion. For this purpose, we used an established murine open-chest model of acute cardiac I/R injury^28,29^. Finally, we further explored the BBG-induced VEGF and Parkin expression in cultured human umbelical endothelial cells (HUVECs) at rest and during acute exposure to hydrogen peroxide (H_2_O_2_), which is an established model of early post-ischemic reperfusion injury *in vitro* ^30^.

## Results

### Effects of P-BBG diet on body weight, food intake and blood glucose levels

**Figure 1:**
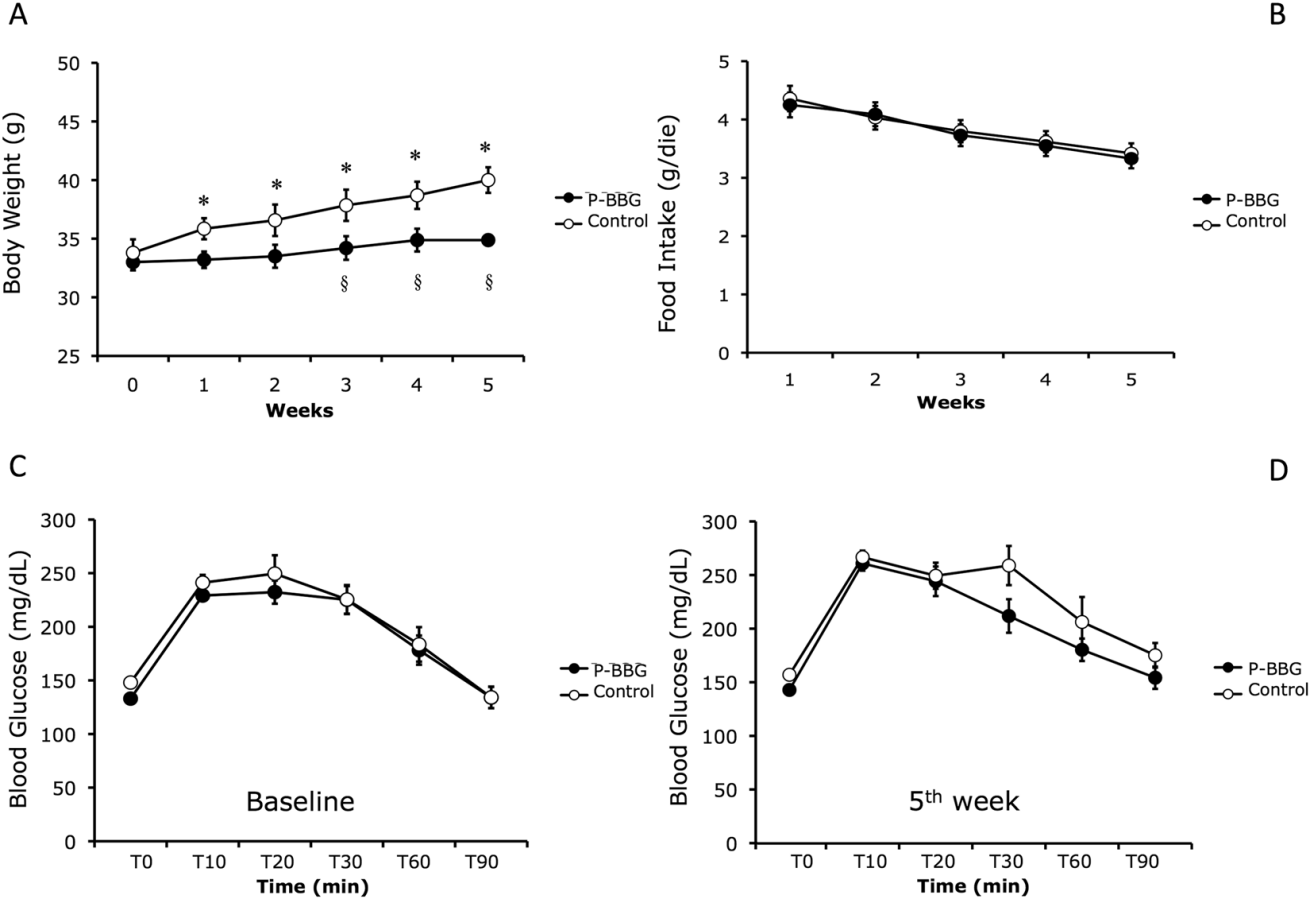
Long-term intake of P-BBG diet prevents body weight gain without altering glucose tolerance. (**A**) Time-dependent effects of diet supplemented with P-BBG (n = 15) or regular pasta (Control; n = 15) on murine body weight. (**B**) Time-dependent changes in food intake in both experimental groups. (**C**) Levels of blood glicemia before (T0) and after intraperitoneal bolus of glucose solution (1 mg/g body weight) at baseline in P-BBG (n = 15) and Control group (n = 15). (**D**) Levels of blood glicemia before (T0) and after intraperitoneal bolus of glucose solution (1 mg/g body weight) after 5 weeks of experimental feeding in both experimental groups. P-BBG: low fat diet supplemented with barley beta-D-glucan enriched pasta (3 g/100 g of dry weight). All measurements are mean ± SD. *p < 0.05 vs. 0 weeks; § p < 0.05 vs. Control.

As shown in Fig. 1A, starting from the baseline, mice fed with the P-BBG diet did not gain weight compared to the Control group. The difference in body weight gain between groups was significant after three, four and five weeks, yet the daily food intake was similar between the two groups (Fig. 1B). At the fifth week of the diet, the basal blood glucose levels (T0) were similar to corresponding values before the diet (baseline) in each experimental group. During the intraperitoneal glucose tolerance test (IPGTT), the profile of blood glucose levels was similar between the two groups before (Fig. 1C) and after (Fig. 1D) the experimental feeding.

### Effects of P-BBG diet on heart rate and cardiac function

**Figure 2:**
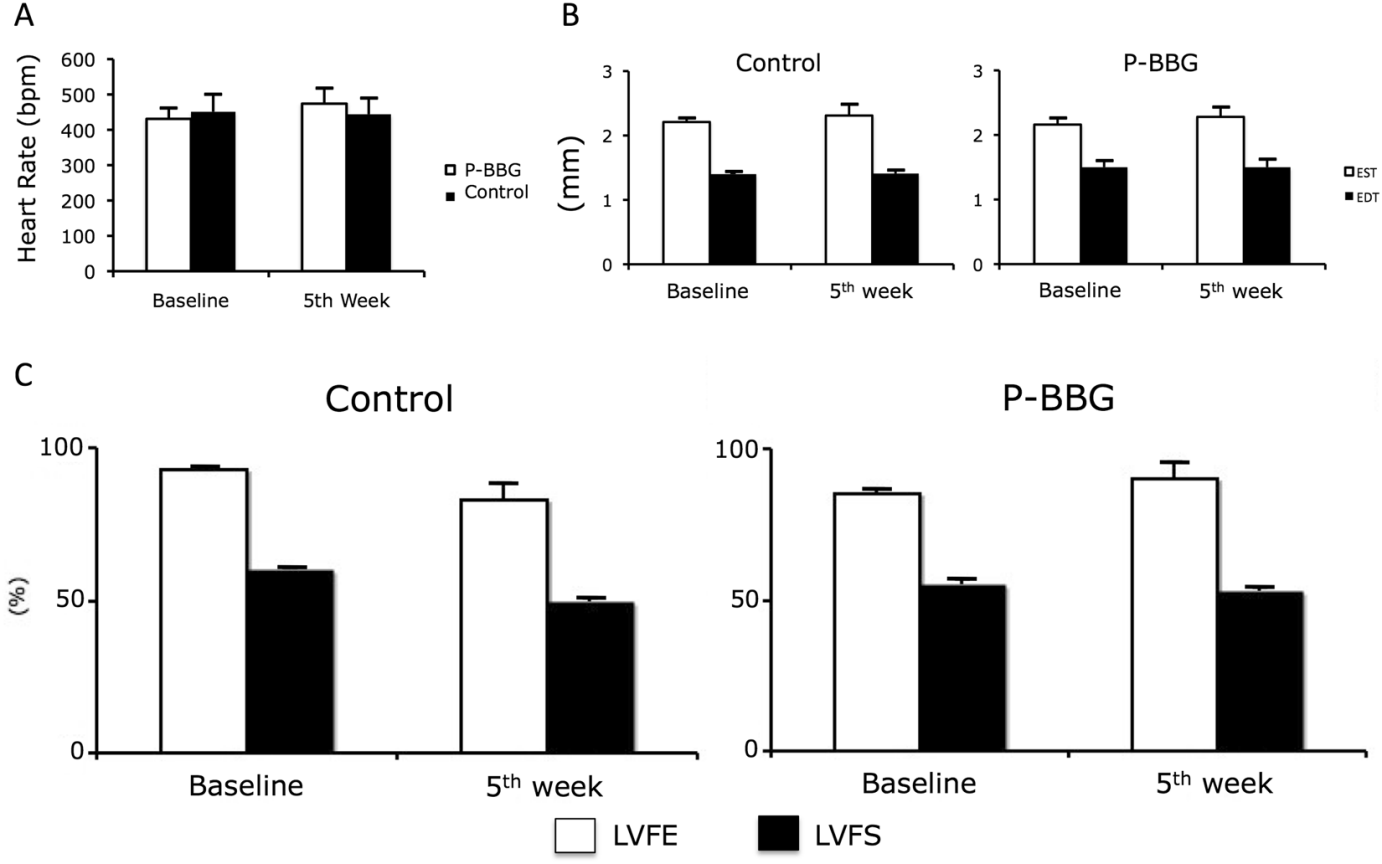
Long-term intake of P-BBG diet does not alter cardiac function. (**A**) The heart rate at baseline and after five weeks of diet is similar in P-BBG (n = 15) and Control (n = 15) mice. (**B**) The sustained intake of diet supplemented with P-BBG does not alter both the end-systolic and end-diastolic left ventricular (LV) wall thickness, which are similar to Control mice. (**C**) The indexes of global LV function are not altered by diet in each experimental group. P-BBG: low fat diet supplemented with barley beta-D-glucan enriched pasta (3 g/100 g of dry weight); EST: end-systolic thickness; EDT: end-diastolic thickness; LVEF: left ventricular ejection fraction; LVFS: left ventricular fractional shortening. All measurements are mean ± SD.

As shown in Fig. 2, after five weeks of regular feeding, the P-BBG diet did not adversely affect the heart rate, the left ventricular (LV) end-diastolic wall thickness, and the LV systolic function of mice.

### Effects of P-BBG diet on survival, infarct scar size and myocardial structure after left ventricular ischemia/reperfusion injury

**Figure 3:**
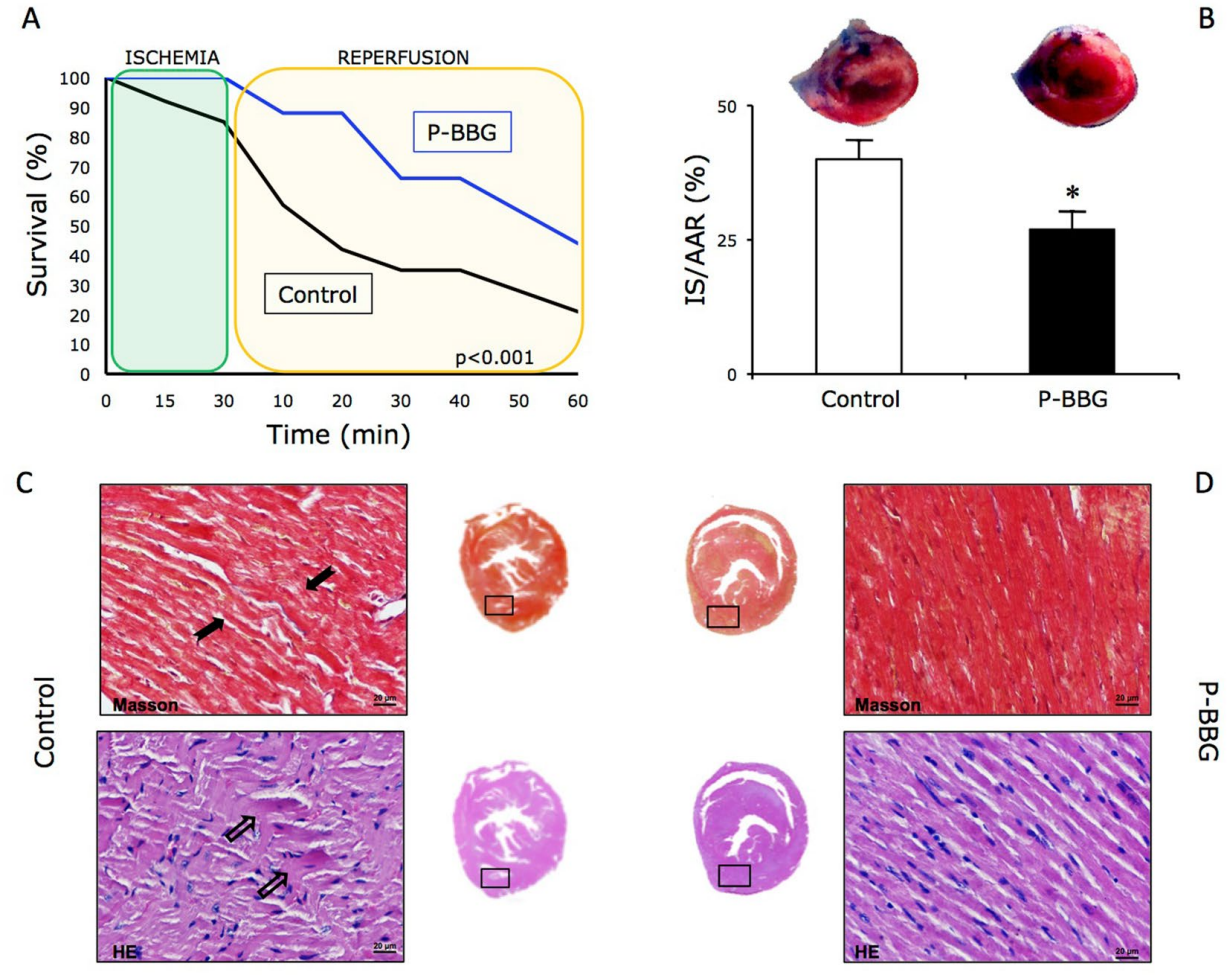
Effects of long-term intake of P-BBG diet on left ventricular ischemia/reperfusion injury. (**A**) The survival rate of mice chronically fed with P-BBG diet (n = 15) exposed to cardiac ischemia/reperfusion injury is significantly higher than Control (n = 15) mice. (**B**) P-BBG diet reduced the left ventricular (LV) infarct size compared to Control mice. The LV infarct size was quantified as a percentage of the ischemic area at risk (IS/AAR (%)) (see methods section). (**C**) Effects of Control diet on the occurrence of contraction band necrosis in Masson (black arrows) and in hematoxylin-eosin (transparent arrows) staining of LV tissue. (**D**) Effects of P-BBG diet on the occurrence of contraction band necrosis in Masson and in hematoxylin-eosin staining of LV tissue. P-BBG: low fat diet supplemented with barley beta-D-glucan enriched pasta (3 g/100 g of dry weight); IS/AAR: infarct size/area at risk; HE: hematoxylin-eosin. All measurements are mean ± SD. *p < 0.05 vs Control.

As shown in Fig. 3A, 44% of the mice fed with the P-BBG diet survived after one hour of post-ischemic reperfusion, whereas only 21% of the Control animals survived after a similar injury at the same time. Hearts harvested from Control mice that had undergone I/R, showed a larger LV necrotic infarct area than those collected from infarcted P-BBG mice (Fig. 3B). The Masson and hematoxylin-eosin staining of LV tissue revealed contraction band necrosis in the hearts of Control mice (Fig. 3C), but not in treated mice (Fig. 3D).

### Effects of P-BBG diet on capillary and arteriolar density, VEGF and dectin-1 protein levels

**Figure 4:**
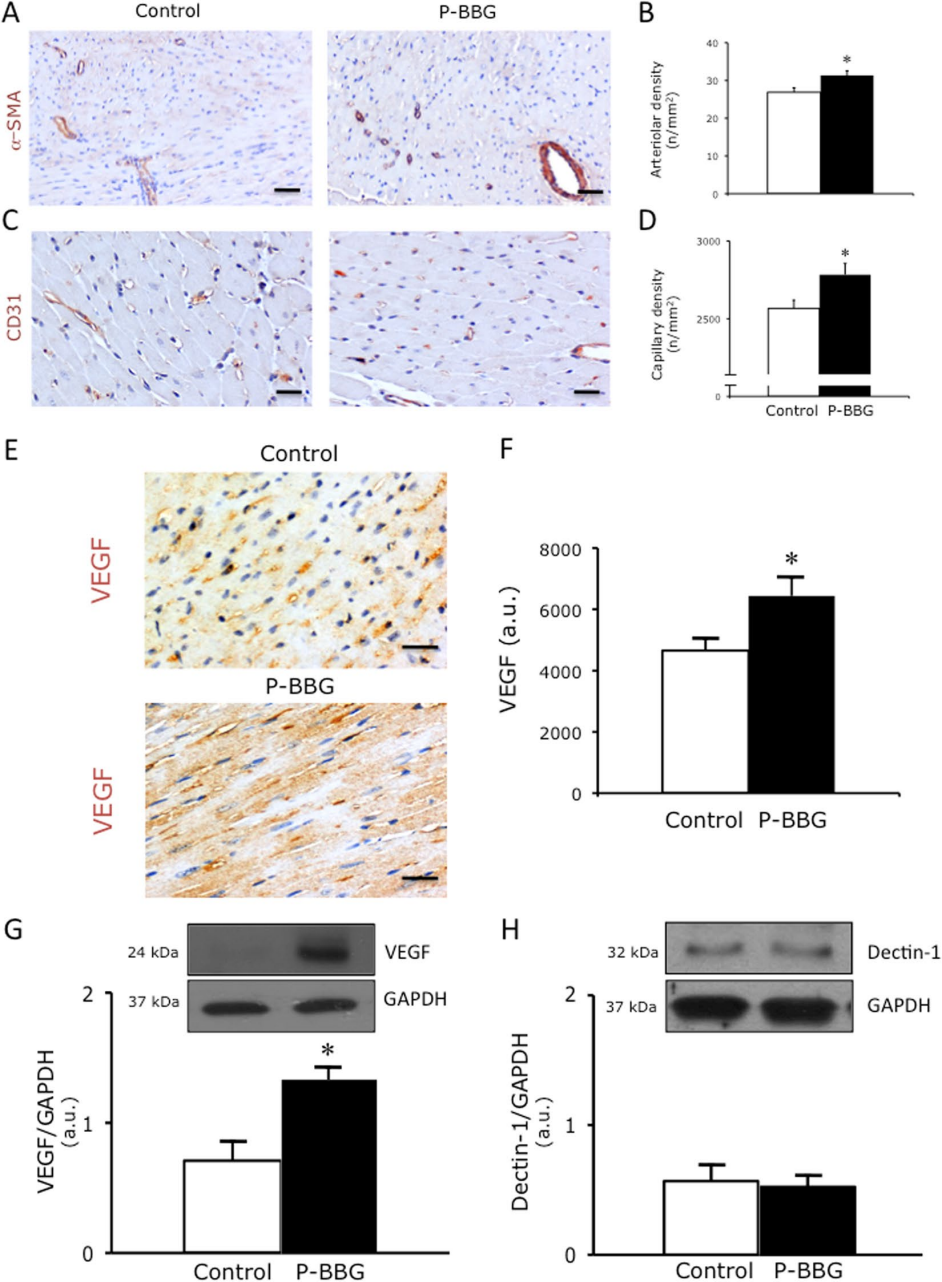
Effects of long-term intake of P-BBG diet on left ventricular capillary and arteriolar density, VEGF and dectin-1 protein levels. (**A**) Representative images of left ventricular (LV) coronary arterioles stained with α–smooth muscle actin in Control and P-BBG hearts. (**B**) The P-BBG diet (n = 15) increased the number of LV coronary arterioles compared to Control group (n = 15); the results are shown as number of arterioles per mm^2^. (**C**) Representative images of LV coronary capillaries stained with CD31 in Control and P-BBG hearts. (**D**) The P-BBG diet (n = 15) increased the number of LV coronary capillaries compared to Control diet (n = 15); the results are shown as number of capillaries per mm^2^. (**E**) Representative images of myocardial VEGF detected in Control and P-BBG left ventricles. (**F**) The P-BBG diet (n = 15) determined a detectable elevation of myocardial VEGF compared to Control diet (n = 15); the results are shown as arbitrary units of intense VEGF staining. (**G**) P-BBG diet (n = 7) increased the myocardial protein expression of VEGF compared to Control diet (n = 7). Levels of VEGF are expressed as arbitrary units of VEGF (24 kDa, MW)/GAPDH (37 kDa, MW) ratio. Representative images of cropped densitometric bands of VEGF are shown. The full-length blots/gels are presented in Supplementary Figure 1A. (**H**) Representative images of cropped densitometric bands of dectin-1 are shown. The full-length blots/gels are presented in Supplementary Figure 1B. The myocardial protein levels of dectin-1 are similar in both Control (n = 7) and P-BBG (n = 7) hearts. Levels of dectin-1 are expressed as arbitrary units of dectin-1 (28 kDa, MW)/GAPDH (37 kDa, MW) ratio. P-BBG: low fat diet supplemented with barley beta-D-glucan enriched pasta (3 g/100 g of dry weight); α-SMA: α–smooth muscle actin; CD31: cluster of differentiation 31; VEGF: vascular endothelial growth factor; GAPDH: glyceraldehyde 3-phosphate dehydrogenase. All measurements are mean ± SD. *p < 0.05 vs. Control.

The myocardial capillary (Fig. 4A–B) and arteriolar (Fig. 4C–D) density of the P-BBG mice was significantly higher than that of the Control hearts. As shown in panels E and F of Fig. 4, cardiac VEGF was considerably more evident in P-BBG than in the Control mice. Similarly, the VEGF protein levels of P-BBG hearts were significantly higher than those of the Control hearts (Fig. 4G). Higher levels of VEGF did not affect the myocardial expression of dectin-1, whose protein levels were similar in both groups (Fig. 4H).

### Effects of P-BBG diet on levels of myocardial oxidative stress, cleaved caspase-3, HIF1-α, pAkt/Akt, pSTAT3/STAT3 and pNF-kB/NF-kB

**Figure 5:**
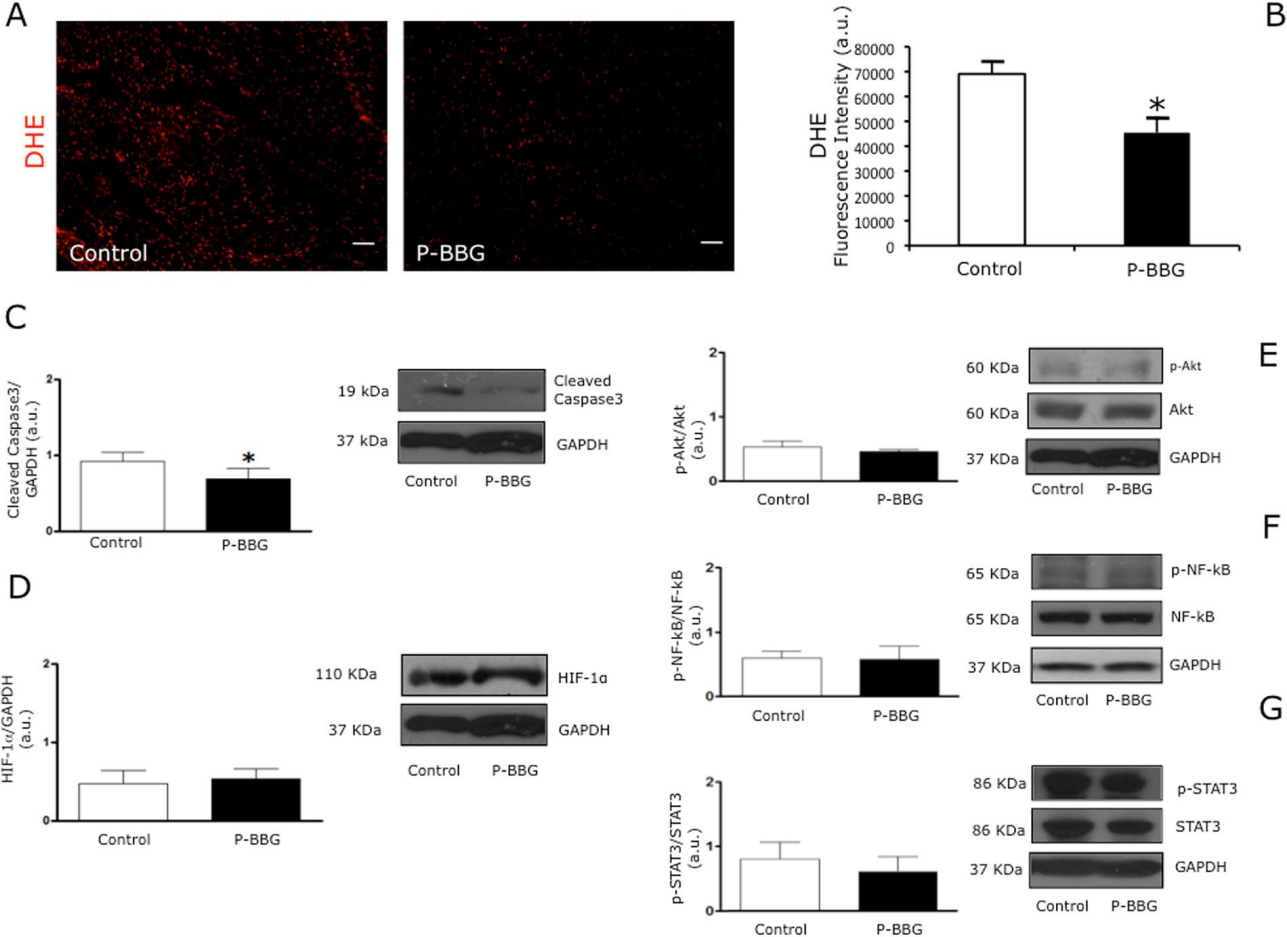
Effects of P-BBG diet on levels of myocardial oxidative stress, cleaved caspase-3, HIF-1α, pAkt/Akt, pSTAT3/STAT3 and pNF-kB/NF-kB. (**A**) Representative images of DHE (dihydroethidium) staining of Control and P-BBG left ventricles. (**B**) P-BBG diet (n = 15) prevented an increase in anion superoxide generation following cardiac ischemia/reperfusion injury compared to Control diet (n = 15); the results are shown as arbitrary units of fluorescence intensity (see Methods). (**C**) The myocardial levels of cleaved caspase-3 were lower in P-BBG (n = 7) than Control (n = 7) hearts. Levels of cleaved caspase-3 are expressed as arbitrary units of cleaved caspase-3 (19 kDa, MW)/GAPDH (37 kDa, MW) ratio. Representative images of cropped densitometric bands of cleaved caspase 3 AND GAPDH are shown. The full-length blots/gels are presented in Supplementary Figure 1C. (**D**) The myocardial levels of HIF-1α are similar in P-BBG (n = 7) and Control (n = 7) hearts. Levels of HIF-1α are expressed as arbitrary units of HIF-1α (110 kDa, MW)/GAPDH (37 kDa, MW) ratio. Representative images of cropped densitometric bands of HIF-1α and GAPDH are shown. The full-length blots/gels are presented in Supplementary Figure 1C. (**E**) The myocardial levels of p-Akt/Akt ratio are similar in P-BBG (n = 7) and Control (n = 7) hearts. Levels of p-Akt (60 kDa, MW)/Akt (60 kDa, MW), normalized to GAPDH levels, are expressed as arbitrary units. Representative images of cropped densitometric bands of p-Akt, Akt and GAPDH are shown. The full-length blots/gels are shown in Supplementary Figure 1C. (**F**) The myocardial levels of p-NF-kB/NF-kB ratio are similar in P-BBG (n = 7) and Control (n = 7) hearts. Levels of p-NF-kB (65 kDa, MW)/NF-kB (65 kDa, MW), normalized on GAPDH levels, are expressed as arbitrary units. Representative images of cropped densitometric bands of p-NF-kB, NF-kB and GAPDH are shown. The full-length blots/gels are presented in Supplementary Figure 1D. (**G**) The myocardial levels of p-STAT3/STAT3 ratio are similar in P-BBG (n = 7) and Control (n = 7) hearts. Levels of p-STAT3 (86 kDa, MW)/STAT3 (86 kDa, MW), normalized on GAPDH levels, are expressed as arbitrary units. Representative images of cropped densitometric bands of p-STAT3, STAT3 and GAPDH are shown. The full-length blots/gels are presented in Supplementary Figure 1E. P-BBG: low fat diet supplemented with barley beta-D-glucan enriched pasta (3 g/100 g of dry weight); HIF-1α: hypoxia-inducible factor 1-alpha; GAPDH: glyceraldehyde 3-phosphate dehydrogenase; p-Akt: phospho-Akt; p-NF-kB: phospho- nuclear factor Kappa b; p-STAT3: phospho- signal transducer and activator of transcription 3. All measurements are mean ± SD. *p < 0.05 vs. Control.

As shown in panels A and B of Fig. 5, the myocardial production of anion superoxide in response to I/R insult was significantly reduced in P-BBG compared to the Control hearts. At one hour of post-ischemic reperfusion, the myocardial cleavage of caspase-3 in the P-BBG hearts was markedly attenuated compared to the Control hearts (Fig. 5C) in the presence of similar protein levels of hypoxia-inducible factor 1-alpha (HIF1-α) (Fig. 5D). The phospho(p)Akt/Akt (Fig. 5E), pSTAT3(signal transducer and activator of transcription 3)/STAT3 (Fig. 5F) and pNF-kB(nuclear factor Kappa b)/NF-kB (Fig. 5G) ratios were similar in both experimental groups.

### Effects of P-BBG diet on myocardial levels of p53 and Parkin

**Figure 6:**
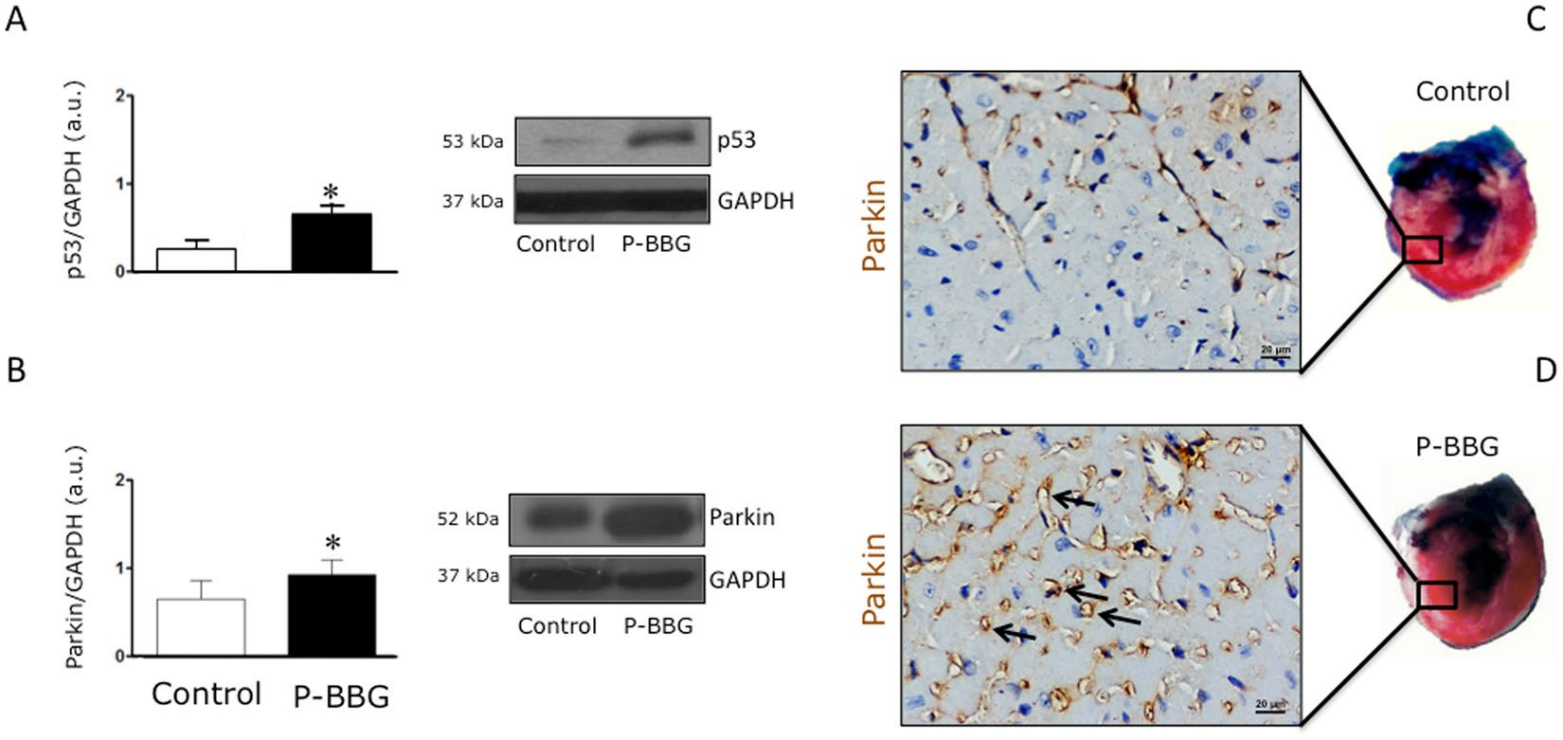
Effects of P-BBG diet on levels of myocardial p53 and Parkin. (**A**) P-BBG diet (n = 7) increased the myocardial protein levels of p53 compared to Control (n = 7) diet. Levels of p53 are expressed as arbitrary units of p53 (53 kDa, MW)/GAPDH (37 kDa, MW) ratio. Representative images of cropped densitometric bands of p53 and GAPDH are shown. The full-length blots/gels are presented in Supplementary Figure 1F. (**B**) P-BBG diet (n = 7) increased the myocardial protein levels of Parkin compared to Control diet (n = 7). Levels of Parkin are expressed as arbitrary units of Parkin (52 kDa, MW)/GAPDH (37 kDa, MW) ratio. Representative images of cropped densitometric bands of Parkin and GAPDH are shown. The full-length blots/gels are presented in Supplementary Figure 1G. (**C**) Representative images of Parkin localization in coronary endothelium of Control left ventricles. (**D**) Representative images of Parkin localization in coronary endothelium of P-BBG left ventricles. P-BBG: low fat diet supplemented with barley beta-D-glucan enriched pasta (3 g/100 g of dry weight); GAPDH: glyceraldehyde 3-phosphate dehydrogenase. All measurements are mean ± SD. *p < 0.05 vs. Control.

As shown in Fig. 6A, the myocardial protein expression of p53 was higher in P-BBG than the Control mice. Conversely, Parkin protein levels were significantly upregulated in P-BBG compared with the Control hearts (Fig. 6B). Although Parkin was weakly detectable in the Control hearts (Fig. 6C), its expression was highly focused in the coronary endothelial cells of the P-BBG hearts (Fig. 6D).

### Acute oxidative stress does not affect the levels of H4 histone acetylation, VEGF and MnSOD in viable endothelial cells treated with barley β-D-glucan

**Figure 7:**
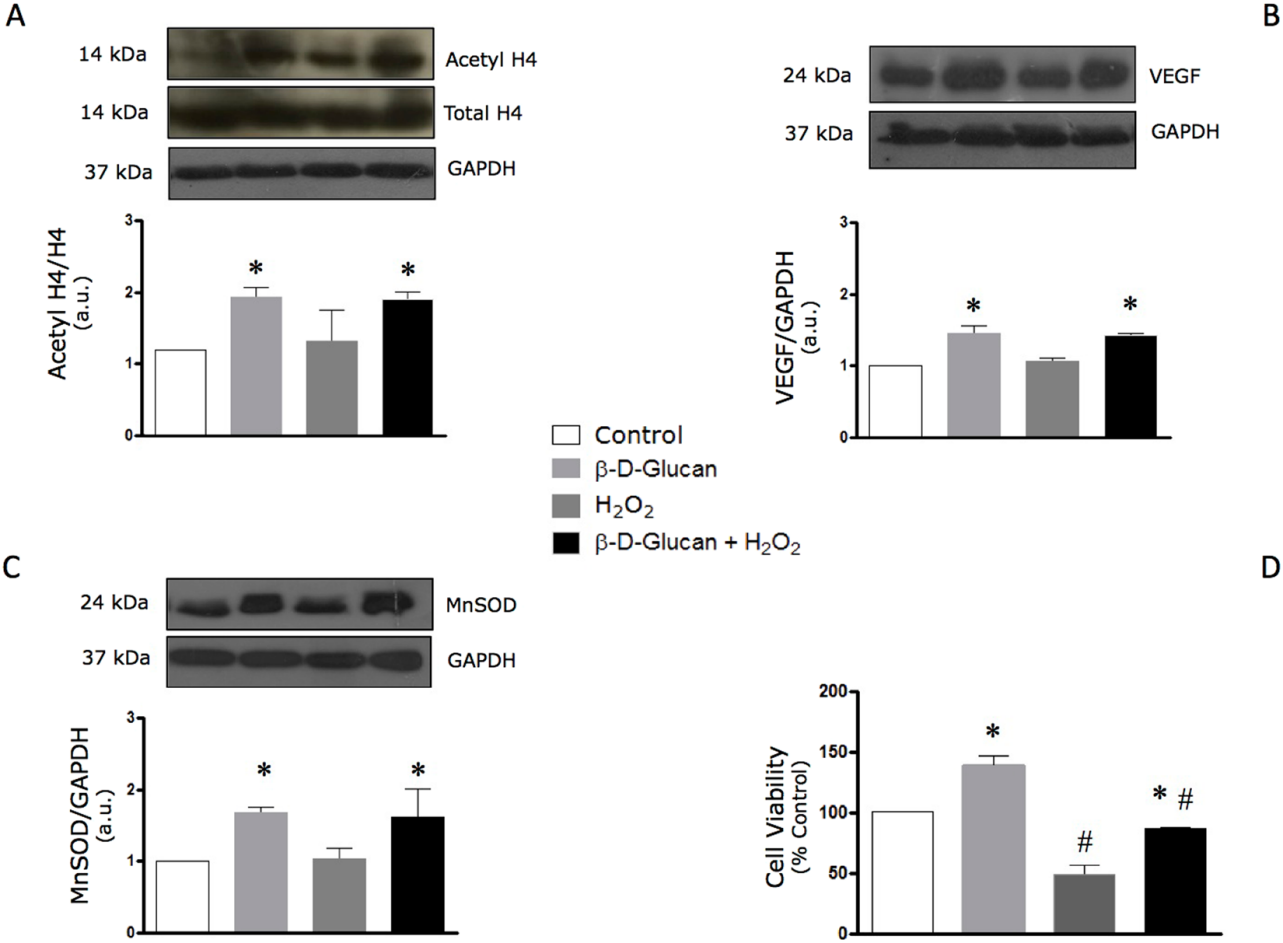
Acute oxidative stress does not affect levels of H4 histone acetylation, VEGF and MnSOD in viable endothelial cells chronically treated with barley β-D-glucan. (**A**) 7-day treatment with 3% barley β-D-glucan (BBG) increases the endothelial levels of acetyl H4 histone compared to untreated HUVECs, which are not affected by acute oxidative stress. Levels of acetyl H4 (14 kDa, MW)/H4 (14 kDa, MW), normalized on GAPDH levels, are expressed as arbitrary units. Representative images of cropped densitometric bands of acetyl H4, H4 and GAPDH are shown. The full-length blots/gels are presented in Supplementary Figure 2A. (**B**) The protein levels of VEGF in HUVECs increase after long-term exposure to 3% BBG compared to untreated cells at rest and during acute oxidative stress. Levels of VEGF are expressed as arbitrary units of VEGF (24 kDa, MW)/GAPDH (37 kDa, MW) ratio. Representative images of cropped densitometric bands of VEGF and GAPDH are shown. The full-length blots/gels are presented in Supplementary Figure 2B. (**C**) The protein levels of MnSOD in HUVECs are increased after long-term exposure to 3% BBG compared to untreated cells, yet are not altered after 1 h exposure to hydrogen peroxide (see Methods). Levels of MnSOD are expressed as arbitrary units of MnSOD (24 kDa, MW)/GAPDH (37 kDa, MW) ratio. Representative images of cropped densitometric bands of MnSOD and GAPDH are shown. The full-length blots/gels are presented in Supplementary Figure 2C. (**D**) BBG promotes the endothelial cell survival after 1 hour treatment with 400 μM H_2_O_2_. HUVECs: human umbelical vascular endothelial cells; H4: histone H4; VEGF: vascular endothelial growth factor; MnSOD: manganese superoxide dismutase; GAPDH: glyceraldehyde 3-phosphate dehydrogenase; H_2_O_2_: hydrogen peroxide. All measurements are mean ± SD. *p < 0.05 vs. untreated condition; ^#^p < 0.05 vs. resting condition.

As shown in Fig. 7, the BBG-induced increase in endothelial levels of H4 histone acetylation (panel A), VEGF (panel B) and MnSOD (panel C) were not altered during the transient exposure of HUVECs to exogenous H_2_O_2_. The decay of cell viability following the acute exposure to hydrogen peroxide was significantly preserved by long-term treatment with BBG (panel D).

### BBG induces the protein expression of Parkin independently of p53 activity

**Figure 8:**
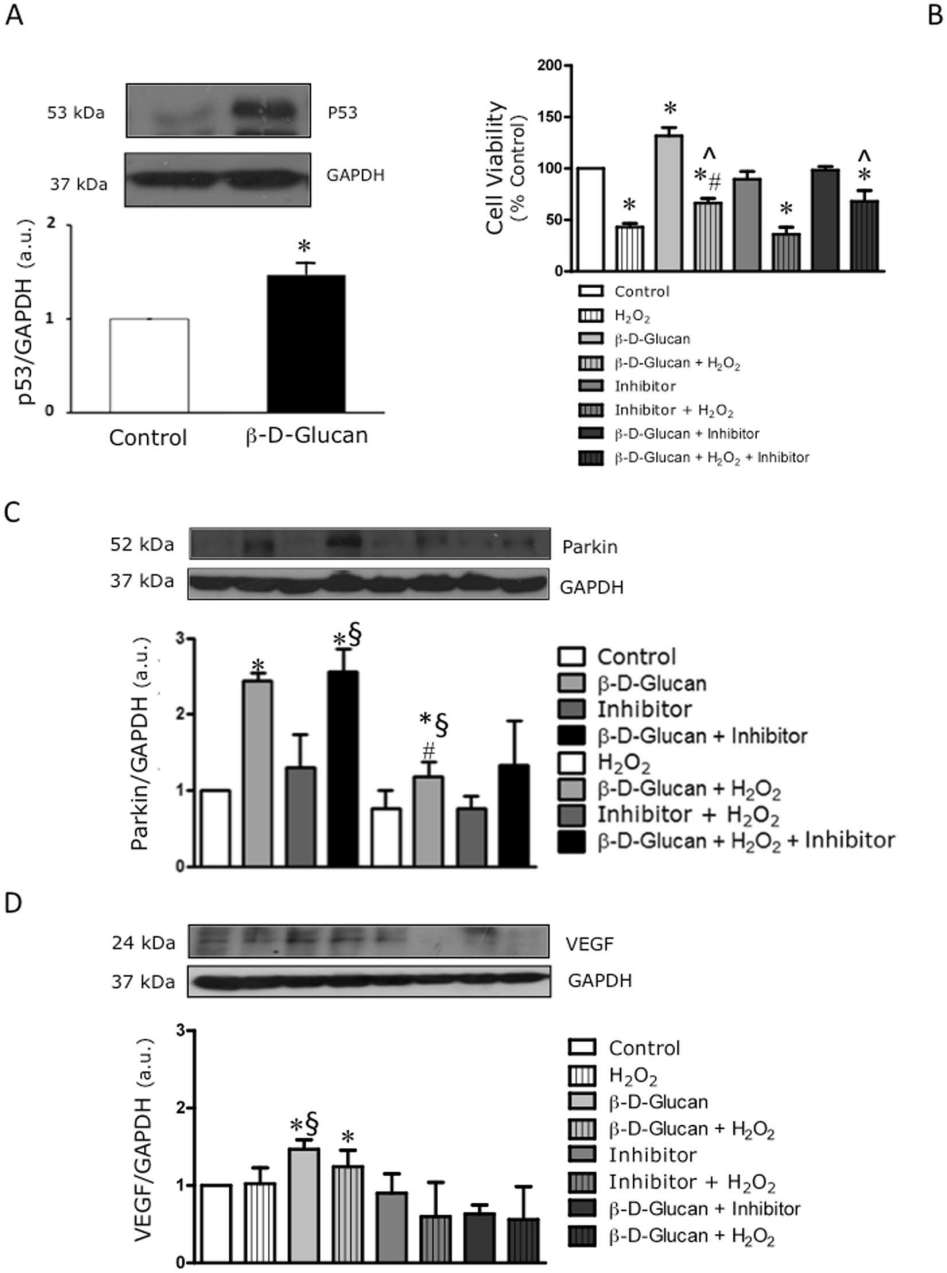
BBG induces the protein expression of Parkin independently of p53 activity. (**A**) 7-day treatment with 3% barley β-D-glucan (BBG) increases the endothelial levels of p53 compared to untreated HUVECs. Levels of p53 are expressed as arbitrary units of p53 (53 kDa, MW)/GAPDH (37 kDa, MW) ratio. Representative images of cropped densitometric bands of p53 and GAPDH are shown. The full-length blots/gels are presented in Supplementary Figure 2D. (**B**) The sustained co-treatment of HUVECs with 3% BBG and PFT-α (Inhibitor) does not impair cell viability. BBG preserves the viability of H_2_O_2_-stressed cells, but not Inhibitor. (**C**) The sustained treatment of HUVECs with 3% BBG increases the protein levels of Parkin compared to untreated cells (Control). The co-treatment with PFT-α does not affect the BBG-induced protein expression of Parkin. In addition, the decay of Parkin protein expression in stressed BBG-treated cells is smaller than in stressed untreated cells. (**D**) HUVECs co-treated with 3% BBG and PFT-α show lower protein levels of VEGF. GAPDH: glyceraldehyde 3-phosphate dehydrogenase; PFT-α: pifithrin-alpha; H_2_O_2_: hydrogen peroxide; VEGF: vascular endothelial growth factor. All measurements are mean ± SD. *p < 0.05 vs. untreated condition; ^§^p < 0.05 vs. Inhibitor; ^#^p < 0.05 vs. corresponding resting condition; ^p < 0.05 vs H_2_O_2_.

The interplay between p53, VEGF and Parkin governs tissue homeostasis through the modulation of angiogenesis^31^ and cellular tolerance to stress^32^. BBG increases the expression of p53 (Fig. 8A) without affecting cell viability, but preserving the cell viability under H_2_O_2_ (Fig. 8B). Although pifithrin-alpha (PFT-α), a well-known inhibitor of p53 activity, does not affect cell viability in resting conditions, it is unable to protect the cells against acute oxidative stress (Fig. 8B). Interestingly, the BBG-induced increase in protein levels of Parkin is not affected by PFT-α. In line with the BBG effects on the viability of H_2_O_2_-stressed cells during co-exposure to PFT-α (Fig. 8B), BBG attenuates the decay of Parkin protein levels in HUVECs exposed to acute oxidative stress independently of p53 activity (Fig. 8C). Conversely, the inhibition of p53 activity by PFT-α counteracts the BBG-induced VEGF expression in HUVECs at rest and during stress (Fig. 8D).

### BBG prevents the rise of anion superoxide levels in a p53-dependent manner

**Figure 9:**
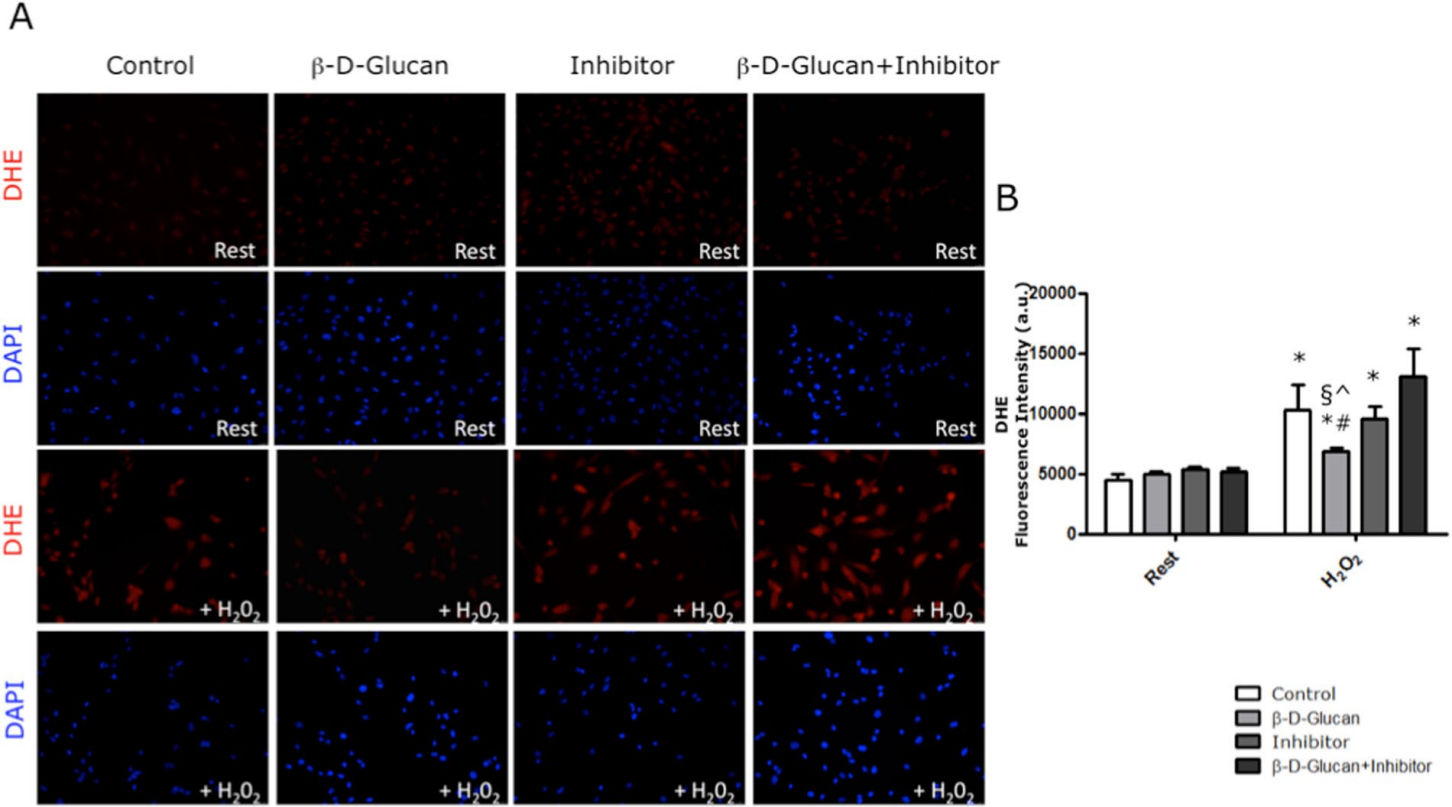
BBG prevents the rise of anion superoxide levels in a p53-dependent manner. (**A**) Representative images of DHE (dihydroethidium) staining of HUVECs at rest and after acute oxidative stress (+H_2_O_2_). (**B**) At rest, the 7-day treatment with 3% BBG (β-D-Glucan), PTF-α (Inhibitor) and β-D-Glucan + Inhibitor did not increase the anion superoxide levels in untreated HUVECs (Control). At stress (+H_2_O_2_), β-D-Glucan prevented an increase in anion superoxide generation compared to untreated cells (Control). Conversely, the 7-day co-treatment with β-D-Glucan + Inhibitor precluded the anti-oxidant effects of BBG in cells exposed to acute oxidative stress. The results are shown as arbitrary units of fluorescence intensity (see Methods). PFT-α: pifithrin-alpha; H_2_O_2_: hydrogen peroxide. All measurements are mean ± SD. *p < 0.05 vs. corresponding resting condition; ^#^p < 0.05 vs. Control + H_2_O_2_; ^§^p < 0.05 vs. Inhibitor + H_2_O_2_; ^^^p < 0.05 vs. β-D-Glucan + Inhibitor + H_2_O_2_.

BBG precludes the rise of anion superoxide levels in H_2_O_2_-stressed endothelial cells, as previously reported by us^22^. HUVECs co-treated for seven days with BBG and non-toxic concentration of PFT-α do not show anion superoxide levels higher than Control cells (Fig. 9A). Interestingly, the inhibition of p53 activity by PFT-α counteracts the BBG-induced reduction of anion superoxide levels in HUVECs exposed to acute oxidative stress (Fig. 9B).

## Discussion

Our study demonstrates that the long-term supplementation of a low fat diet with pasta enriched with BBG increases the survival rate of mice undergoing LV I/R injury. The magnitude of the LV infarct scar size and of the contraction necrotic bands was safely attenuated in mice fed with P-BBG for five weeks. The dietary intake of P-BBG also prevented an increase in body weight without altering the systemic glucose tolerance, cardiac function, and the well-being of mice. Compared to the Control group, the histological analysis revealed a higher density of coronary capillaries and arterioles in the explanted hearts of P-BBG mice, which support higher tissue viability downstream of the obstructed coronary tree. Regarding the cardioprotective pathways, our data revealed, for the first time, the relationship between myocardial VEGF, p53 and Parkin protein upregulation, vasculogenesis enhancement and reduction of caspase-3 cleavage at the early stages of post-ischemic reperfusion conferred by the sustained dietary intake of P-BBG.

It is largely recognised that cardiac ischemic preconditioning enhances the myocardial protein expression of VEGF^33^, a growth factor that induces myocardial angiogenesis and cell survival^34^. In line with the cardioprotective effects of VEGF, other researchers have perfused the heart with VEGF^35^ or have upregulated the myocardial VEGF expression using gene therapy^36,37^ in order to enhance the local adaptive response naturally occurring under oxidative microenvironment. However, none of these invasive approaches may be ethically permissible in healthy subjects. Hence, the urgent need to develop a noninvasive strategy to enhance collateral artery growth in the adult myocardium and to promote the survival of cardiac cells following I/R injury.

In a previous study, we demonstrated that chronic exposure of endothelial cells to water-soluble BBG enhances the pro-angiogenic and pro-survival expression of manganese superoxide dismutase (MnSOD)^22^, which is a mediator of ischemic preconditioning^38^.

In the current study, we thus hypothesized that the daily dietary intake of water-soluble BBG might promote the formation of new coronary vessels in healthy subjects and prevent myocardial I/R injury.

For this purpose, healthy mice were fed with a low fat diet supplemented with pasta made with a mixture of barley flour and wheat flour, which guarantees a high content of viscous polysaccharides and a regular BBG intake. Our hypothesis is supported by the evidence that pasta safely provides a BBG-enriched diet^27,39^. To the best of our knowledge, no previous study has assessed the effects of diet supplemented with BBG-enriched pasta to counteract the onset of myocardial I/R injury.

First, we have observed that P-BBG-enriched diet precludes gain weight in mice. Our data are consistent with recent clinical study showing that daily intake of barley beta-D-Glucan prevents visceral fat obesity and body weight gain in humans^40^. In fact, the ability of soluble BBG to form highly viscous solutions in the human gut slows gastric emptying, digestion and absorption of dietary fat^41^.

In our study, the survival rate at an early stage of post-ischemic reperfusion was significantly higher in mice fed with P-BBG than with the wheat pasta. Compared to the Control group, the IS/AAR ratio revealed that the left ventricles of P-BBG mice were less injured and the contraction band necrosis, a hallmark of immediate necrotic cell death during the first minutes of reperfusion^42^, was undetectable in treated hearts. Since myocardial injury is induced after an overnight fasting period, it is conceivable that cardioprotection depends on increased myocardial angiogenesis due to the sustained intake of P-BBG. Our hypothesis is well supported by previous clinical evidence showing that individual tolerance to cardiac post-ischemic reperfusion injury is due to a higher density of coronary collaterals^4,5^.

In line with our hypothesis, the myocardial density of coronary capillaries and arterioles was higher in mice fed with a diet supplemented with P-BBG than with regular pasta. Although the myocardial protein levels of dectin-1, a receptor that mediates endothelial β-D-Glucan effects^43^, were similar in both experimental groups, the marked increase in VEGF protein levels in the P-BBG hearts is noteworthy and is closely linked to the regular intake of BBG and to the weight loss. Indeed, we cannot exclude that BBG-based diet may induce VEGF expression in other tissues. It is known that an increase in VEGF expression in adipose tissue can result in increasing vascular density^44^, in reducing adipocyte size and in precluding gain weight in mice^45,46^.

In addition, the myocardial levels of anion superoxide decreased significantly in the reperfused hearts of mice fed with P-BBG rather than in the Control mice.

These new *in vivo* findings strongly support our previous study^22^. In fact, VEGF likely enhances the local expression of MnSOD^23^, a key anti-oxidant enzyme, which contributes to the angiogenesis^22^ and higher cardiac tolerance against ischemia/reperfusion injury^47^ in P-BBG mice.

Since natural compounds may promote VEGF/antioxidant signaling and inhibit the onset of apoptosis in hearts undergoing I/R^48^, we assessed the myocardial levels of cleaved caspase-3, an established pro-apoptotic factor. The levels of cleaved caspase-3 were significantly lower in P-BBG hearts, even though the myocardial protein expression of HIF-1α, a transcriptional factor involved in the induction of VEGF expression^49^ as well as in the inhibition of apoptosis^50^, was similar in both experimental groups. These results are in agreement with our previous *in vitro* study^22^ and suggest the activation of different signaling pathways by BBG.

Although several studies have found a key role for phosphorylated Akt to mediate the cardioprotective phosphatidylinositide 3-kinase (PI3K) signaling pathway^51^ during preconditioning, the myocardial levels of phospho-Akt/total-Akt ratio were the same in treated and untreated mice.

We then examined the role of the P-BBG diet on myocardial STAT3 signaling pathways since STAT3 activation has been implicated in the inhibition of caspase-3 cleavage^52^ and also in the upregulation of MnSOD^53^ and VEGF^54^ genes. Surprisingly, cardiac STAT3 was also similarly expressed and phosphorylated in both experimental groups.

Lastly, although active NF-kB promotes the VEGF-dependent modulation of MnSOD expression^55^, the myocardial levels of phospho-NF-kB/total-NF-kB ratio were similar in both the P-BBG and Control hearts.

Although the activation of the pathways so far investigated plays a role in regulating VEGF levels during preconditioning, it seems that they are not activated by BBG in our model. Since p53 has been recently considered an important regulator of cardiac gene transcription^56^, we have hypothesized that it may play a key role in mediating the BBG-induced cardioprotection.

Farhang Ghahremani *et al*.^57^ demonstrated that p53 induces the expression of VEGF. In our study, the expression of p53 in P-BBG hearts was higher than in the Control hearts. It is conceivable that p53 leads to VEGF promoter activation by acting synergistically with HIF-1α, as previously demonstrated by others^57^.

We have demonstrated, for the first time, that dietary intake of P-BBG induces myocardial p53 upregulation without promoting systemic toxicity or cardiac cell death. Our findings are not in agreement with a previous study that found that dietary anti-oxidants increase p53 levels to the point where they induce apoptosis^58^. This can be explained by the fact that P-BBG diet also increased the myocardial protein expression of Parkin, an E3 ubiquitin ligase, which plays a key role in promoting mitophagy^59^ and in delaying caspase-3 activation^60^. Since healthy mice were safely fed with the P-BBG-enriched diet for five weeks, our results would seem to suggest that the physiological interplay between higher levels of p53 and Parkin may contribute to the non-invasive regulation of the VEGF expression and myocardial viability status.

As β-Glucan receptors are expressed on the surface of endothelial cells^61^, the upregulation of Parkin protein was localized in the coronary endothelial cells of P-BBG hearts. The binding affinity of dectin-1 to β-D-Glucan and its biological activity are similar in humans and mice^22,62^, it would thus appear that we have revealed BBG-dependent mechanisms in cultured HUVECs at rest and under acute oxidative stress. On day 7 after the exposure of HUVECs to 3% BBG, the levels of acetylated H4 histone, VEGF and MnSOD increased and were not affected by acute oxidative stress. Our *in vitro* results suggest that the acute oxidative burst induced by I/R injury *in vivo* did not counteract the BBG-induced regulatory effects on the abovementioned proteins.

Finally, we have demonstrated that the 7-day treatment of HUVECs with BBG similarly increased the protein expression of p53 and Parkin in the presence of increased histone acetylation. Our data agree with previous studies showing that class I HDAC inhibitors, such as BBG, upregulate the protein expression of p53^63^ and Parkin^64^. As a function of expression levels, p53 can either prevent or promote apoptosis^65^. *In vitro*, however, BBG-induced p53 upregulation did not affect the viability of endothelial cells.

Although the inhibition of p53 activity is essential to enhancing the mitophagic activity of Parkin^66^, it is still unknown whether the VEGF and Parkin protein expression induced by BBG depends on p53 activity. Consequently we performed additional experiments by treating HUVECs with BBG in the presence of PFT-α, an established inhibitor of p53 activity^66^. Although PFT–α did not alter the expression of Parkin, the BBG-induced upregulation of Parkin was not affected by inhibition of p53 activity.

At least, BBG mitigates the H_2_O_2_-induced reduction of Parkin protein expression independently of p53 activity. Hence, our results suggest that the Parkin expression induced by BBG is independent of p53 activity. Conversely, the BBG-induced increase in VEGF protein levels was hampered by PFT–α, which confirms that BBG increased the VEGF expression in a p53-dependent manner. Similarly, PFT–α hindered the BBG-induced decrease in anion superoxide levels in stressed cells, which confirms that BBG decreased the oxidative stress in a p53-dependent manner. Our findings reveal for the first time the impact of BBG on the simultaneous endothelial expression of VEGF and Parkin interacting differently with p53 activity. Moreover, we have demonstrated that the anti-oxidant effects of BBG depends on the p53 activation.

**Figure 10:**
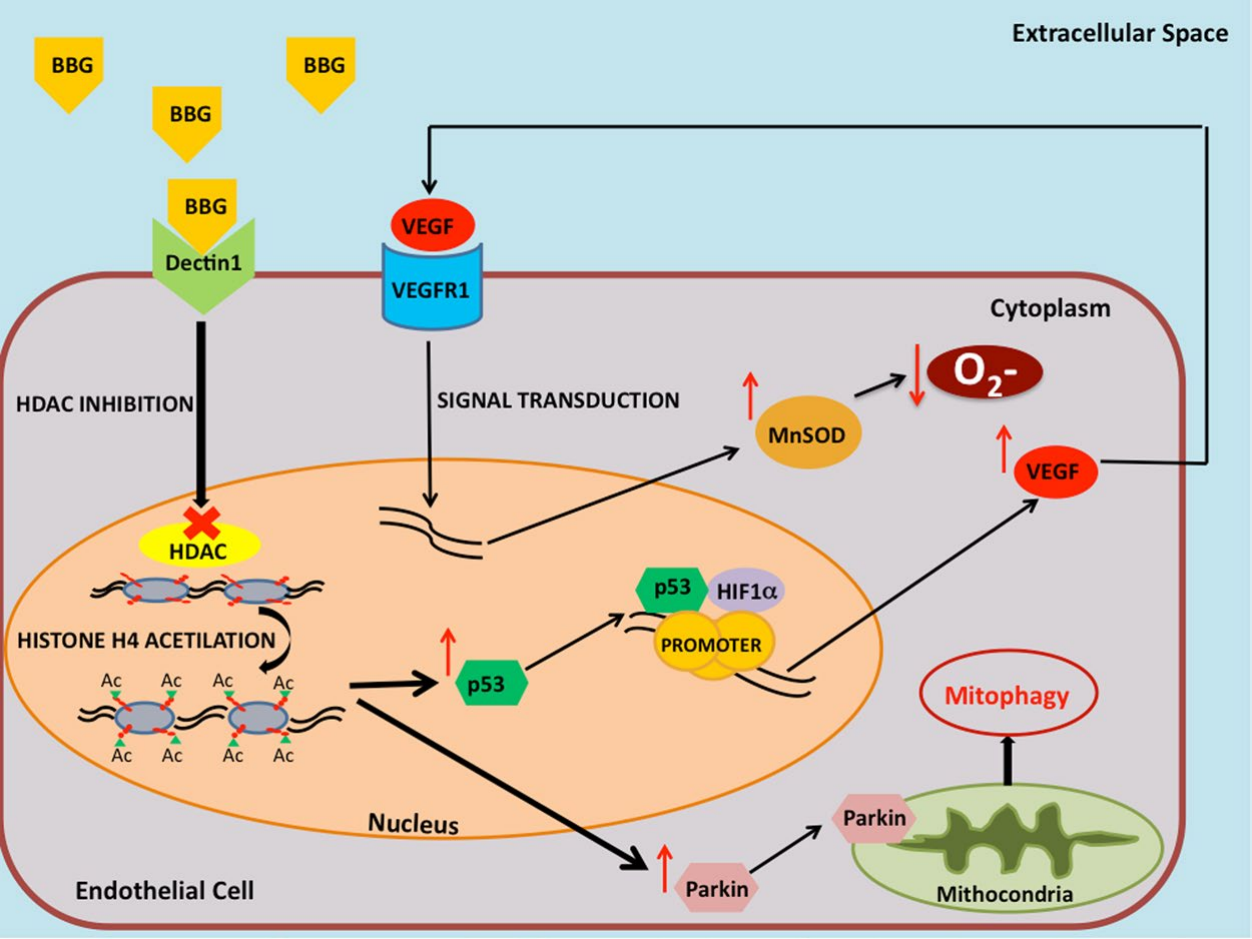
Proposed mechanism of BBG-mediated regulation of endothelial tolerance to ischemia-reperfusion injury The proposed mechanism of BBG-mediate regulation of endothelial tolerance to oxidative stress following ischemia-reperfusion injury through upregulation of VEGF and Parkin. BBG: barley-derived (1.3) β-D-Glucan; HDAC: class I histone deacetylase; Ac: acetyl; VEGF: vascular endothelial growth factor; VEGFR1: vascular endothelial growth factor receptor type 1; HIF-1α: hypoxia-inducible factor 1-alpha; MnSOD: manganese superoxide dismutase; O_2_−: anion superoxide.

Therefore, by enhancing class I HDAC inhibition, the long-term dietary intake of P-BBG may simultaneously promote the neoangiogenesis and higher tolerance to oxidative stress due to enhanced endothelial expression of VEGF/MnSOD signaling and Parkin (the proposed mechanism is summarized in Fig. 10).

## Conclusions

To the best of our knowledge, this is the first study to show *in vivo* data demonstrating that a sustained dietary intake of pasta enriched with BBG safely increases coronary collaterals, limits the infarct size, and reduces mortality. This is achieved through the simultaneous myocardial upregulation of p53, VEGF and Parkin without interfering with the ability of cardiac cells to phosphorylate Akt, STAT3 and NF-kB during the post-ischemic reperfusion. Higher p53, VEGF and Parkin protein levels in endothelial cells chronically treated with BBG are not affected by exposure to acute oxidative stress. Finally, Parkin responsiveness to BBG is not driven by the inhibition of p53 activity, which in turn may contribute to VEGF expression^31^ and anion superoxide decay.

We believe our findings will help in the design a novel nutraceutical approach in the clinical noninvasive prevention of cardiac I/R injury.

## Methods

### Experimental protocol and animal model of left ventricular I/R injury

Thirty adult male C57BL/6 J mice (body weight 22–25 g; 4–8 weeks old) were housed with a constant light and dark phase of 12 hours at 20–23 °C. They were fed for five weeks with a low fat diet (LFD; 3.085 Kcal/g metabolizable energy; 3.5% fat; 17.6% proteins; A. Rieper SpA, BZ, Italy) supplemented with BBG-enriched pasta (3 g/100 g of dry weight) (P-BBG, n = 15) or regular wheat pasta (Control, n = 15), kindly provided by Granoro srl (Corato, Italy). The body weight and food intake of mice were assessed once a week. The composition of the diets is summarized in Table 1.

**Table 1:** Composition of normal diet supplemented with regular or functional pasta.

	Control	P-BBG
Proteins (%)	17.6	17.6
Fat (%)	3.5	3.5
Carbohydrates (%)	60.3	61.6
Ashes (%)	5.5	4.1
Others (%)	3.7	3.7
Calories (Kcal/g)	3.085	3.085

P-BBG, low fat diet supplemented with barley beta-D-glucan enriched pasta (3 g/100 g of dry weight).

At the beginning and the end of the experimental feeding, transthoracic echocardiography^67^ and an intraperitoneal glucose tolerance test^68^ were performed in 6 h fasted mice. At the end of the fifth week of the diet, the mice underwent acute myocardial I/R, as previously described^69^. Briefly, the chest was opened and the left anterior descending coronary artery was transiently ligated at 2 mm lower than the tip of the left auricle in animals anesthetized with isoflurane (1.5–2%) in 100% oxygen with a flow rate of 0.4 L/min until loss of righting reflex, ventilated via a tracheal intubation at a tidal volume of 0.30 ml and respiratory rate of 100 breaths/min (Ugo Basile Srl, Varese, Italy). Body temperature was maintained constant between 36.8 °C and 37 °C by a thermoregulated surgical table connected to a rectal probe (Ugo Basile Srl, Varese, Italy). Thirty minutes of coronary occlusion was followed by reperfusion for 60 min under monitoring of three-lead electrocardiography (ECG). At the end of myocardial reperfusion, the LAD was reoccluded and the phtalocyanine blue dye was injected into the LV cavity to perfuse the nonischemic portions of the myocardium. Then, the heart was rapidly removed for infarct size, histological and molecular assessment. All animal procedures were approved by the Italian Ministry of Health and conducted in conformity with the guidelines from Directive 2010/63/EU of the European Parliament on the protection of animals used for scientific purposes.

### Transthoracic echocardiography

Cardiac function in mice lightly sedated by intraperitoneal injection with Zoletil® (tiletamine 15 mg/kg, zolazepam 15 mg/kg; Virbac, Peakhurst, New South Wales, Australia) was assessed by transthoracic echocardiography using a commercially available ecocardiographer equipped with a 12-MHz probe (MyLab30, Esaote, Italy), as previously described^70^.

Each parameter was measured using B-mode guided M-mode imaging in the parasternal short axis-view at the level of the papillary muscles. Images were analysed offline in a blinded fashion. The global cardiac function was calculated as the percentage ejection fraction of the left ventricle (%LVFE). Heart rate was simultaneously obtained with a three-lead ECG monitor.

### Intraperitoneal glucose tolerance test

The glucose tolerance test was performed as previously described^68^. Briefly, glucose sterile solution was intraperitoneally injected (1 mg/g body weight) in each awake 6 h fasting mouse after baseline blood glucose measurement. The blood glucose levels were measured at 10, 20, 30, 60, and 120 min after bolus glucose.

### Infarct size, histological and immunohistochemical assays

The LV infarct size was assessed as previously described^28^. Each one-millimeter-thick left ventricular transverse section was photographed with a light microscopy (Olympus BX43) at 10X original magnification and digitized by a video system (Olympus DP20 camera) interfaced with a computer with dedicated software (CellSens Dimension, Olympus) for morphometric and/or color analysis. The infarct size (IS; non stained by 1% solution of 2,3,5-triphenyltetrazolium chloride, Sigma-Aldrich) was measured with Image J software, and expressed as percentage of the ischemic area at risk (AAR; non stained by phtalocyanine blue dye, Sigma-Aldrich Chemical Co, MO, USA).

Serial 5-μm frozen heart sections were stained with H&E for overall morphology assessment. The density of the left ventricular coronary arterioles and capillaries stained by immunohistochemistry was quantified as the number of smooth muscle α-actin - (α-SMA, 1:100, Santa Cruz Biotechnology Inc, USA) and/or CD31-positive structures (1:100, Abcam Inc., Cambridge, UK) per mm^2^ using Image J. Finally, to show the cell source of VEGF, additional LV sections were further stained with an anti-VEGF antibody (1:100, Santa Cruz Biotechnology Inc, USA).

### Detection of myocardial anion superoxide generation

We used a dihydroethidium assay (DHE; Sigma-Aldrich Chemical Co, MO, USA) to examine the anion superoxide formation in the frozen heart tissue sections, as previously described^71^. *In vitro*, endothelial superoxide anion generation was determined by staining of HUVECs in each experimental condition as previously described by us^22^. Fluorescence microscopy was performed with a Leica TCS DMIRE 2 (LCS Lite Software; Leica, Wetzlar, Germany).

### Western blot assays

Heart tissues were homogenized in a RIPA buffer containing protease and phosphatase inhibitors (Pierce, Rockford, USA) at high-frequency (25 Hz) with TissueLyser (Quiagen S.p.A, Milan, Italy). Homogenates were centrifuged at 12000 g for 15 minutes at 4 °C to remove nuclei and cell debris. The protein concentration in supernatants was determined using a BCA protein assay kit (Pierce, Rockford, USA). Equal amounts of protein (30 μg) were fractionated by 8–15% SDS polyacrylamide gel and transferred to polyvinylidene difluoride membranes (PVDF, Thermo Scientific, Rockford, USA). Membranes were blocked with 5% non-fat dried milk in TBS/Tween20 (0.01%) at room temperature (RT) for 1 hour, and then probed with primary antibodies at a p-Akt (p-Akt; 1:1000, Cell Signaling, Danvers, MA), total Akt (1:1000, Cell Signaling, Danvers, MA), p-STAT3 (1:1000, Cell Signaling, Danvers, MA) and total STAT3 (1:1000, Cell Signaling, Danvers, MA), which are pro-survival signaling proteins involved in some cardioprotective pathways^72^, Parkin (1:500, Abcam Inc., Cambridge, UK), a key protein mediating mitophagy^59^, p53 (1:1000, Abcam Inc., Cambridge, UK), a potential regulator of Parkin expression^73^, p-NF-kB (1:1000, Cell Signaling, Danvers, MA) and total NF-kB (1:1000, Cell Signaling, Danvers, MA), a transcriptional factor involved in the inflammatory response and VEGF secretion by cardiomyocytes^74^, cleaved-caspase3 (1:1000 Santa Cruz Biotechnology Inc, USA), a pro-apoptotic factor^75^, hypoxia-inducible factor 1-alpha (HIF1-α;1:1000 Abcam Inc., Cambridge, UK), VEGF (1:1000 Santa Cruz Biotechnology Inc, USA), manganese superoxide dismutase, a key antioxidant enzyme (MnSOD, 1:1000, Millipore, MA, USA), and dectin-1 (1:1000 Novus Biologicals, USA), the β-Glucan receptor.

The ratio between pan-acetylated histone H4 type (1:1000, Millipore, MA, USA) and total histone H4 type (1:1000, Abcam Inc., Cambridge, UK) was assessed as previously described^22^. The membranes were reprobed for GAPDH (1:1000, Santa Cruz Biotechnology Inc, USA) to verify the uniformity of protein loading. The phospho/total ratio were calculated using the normalized values. After incubation with the above mentioned antibodies, and rinsing with TBS/Tween20 (0.01%) three times for 10 minutes, membranes were incubated with horseradish peroxidase-conjugated (HRP-conjugated) anti-rabbit or anti-mouse secondary antibodies (Abcam Inc., Cambrige, UK) for one hour at room temperature. Specific protein bands were detected using an ECL Plus Western blot detection system (Bio-Rad Laboratories Inc., CA, USA). Densitometry analysis of protein bands was performed using ImageJ software (National Institute of Healt, USA). We checked the predicted molecular weight trough the use of Precision Plus Protein™ Dual Color Standards (Bio-Rad Laboratories, Inc., Hercules, CA, USA). All Western blot experiments were performed in triplicate.

### Cell lines and *in vitro* experimental protocol

Human umbilical vein endothelial cells (HUVECs, Combrex Bio Science Inc, Walk-ensville, MD, USA) were cultured in EndoGroTM Basal Medium (Millipore, MA, USA) with 10% fetal bovine serum (FBS), 1% penicillin-streptomycin, and 1% L-glutamin. HUVECs were cultured with 3% w/v β-D-glucan (Sigma-Aldrich Chemical Co, MO, USA) or with PBS (negative control) for seven days and then were exposed for 1 h to H_2_O_2_ (Sigma-Aldrich Chemical Co, MO, USA) with a pro-apoptotic dose (400uM). The acute exposure to H_2_O_2_ recapitulates the reperfusion injury^30^. Unstressed cells were used as the Control. In order to evaluate the BBG-dependent regulation of endothelial Parkin protein expression, HUVECs were co-treated for seven days with BBG and non-toxic concentration of PFT-α (10 uM; Sigma-Aldrich Chemical Co, MO, USA), a p53’s mitochondrial translocation inhibitor^76^.

### MTT assay

After 1 h exposure to H_2_O_2_ or PBS, the cell viability was assessed using the MTT reduction assay (Sigma-Aldrich Chemical Co, MO, USA) according to the manufacturer’s instructions and as previously described by us^77^.

### Statistical analysis

Data are presented as mean ± SD. One-way and two-way ANOVA followed by Bonferroni post hoc test were used to compare multiple groups. P < 0.05 was set as a threshold for statistical significance. Statistical analysis was performed using SPSS for Windows, version 11.1 (SPSS, Chicago, IL). The survival curves were developed using the Kaplan-Meier function with the logrank test (GraphPad Prism 4.0).

## Electronic supplementary material

Supplementary Informations
