# Supplementary material for: Long-term Intake of Pasta Containing Barley (1–3)Beta-D-Glucan Increases Neovascularization-mediated Cardioprotection through Endothelial Upregulation of Vascular Endothelial Growth Factor and Parkin
Source: Sci Rep. 2017 Oct 18;7:13424. doi: 10.1038/s41598-017-13949-1 (PMC5647408; doi:10.1038/s41598-017-13949-1)
Supplement: Supplementary file 1 — Supplementary Informations [file 41598_2017_13949_MOESM1_ESM.doc]

**SUPPLEMENTARY FILES**

**Long-term Intake of Pasta Containing Barley (1-3)Beta-D-Glucan Increases Neovascularization-mediated Cardioprotection through Endothelial Upregulation of** **Vascular Endothelial Growth Factor and Parkin**

Valentina Casieri^1^, Marco Matteucci^1^, Claudia Cavallini^2^, Milena Torti^3^, Michele Torelli^3^, Vincenzo Lionetti^1,4^

^1^ Institute of Life Sciences, Scuola Superiore Sant'Anna, Pisa, Italy

^2^ Laboratory of Molecular Biology and Stem Cell Engineering, National Institute of

Biostructures and Biosystems, Bologna, Italy.

^3^ Research and Development Unit, Pastificio Attilio Matromauro Granoro s.r.l., Corato,

Italy.

^4^ UOS Anesthesia and Intensive Care, Fondazione Toscana “G. Monasterio”, Pisa, Italy

Supplemental Figure 1


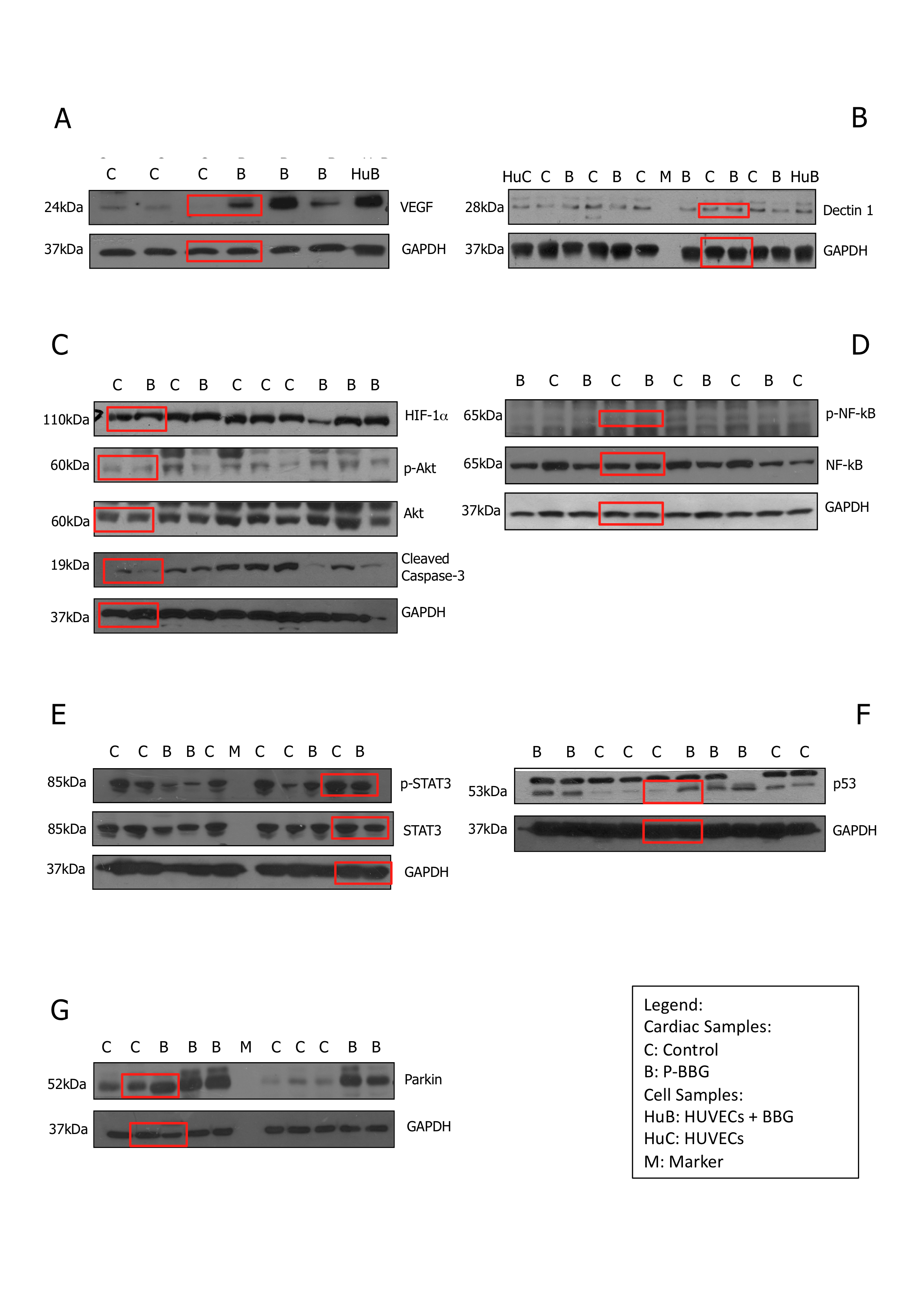


**Supplementary Figure 1:** Western blots assay of cardiac tissues. Panel A, B, C, D, E, F, G show the full-length Western blots corresponding to cropped blots in the main text.


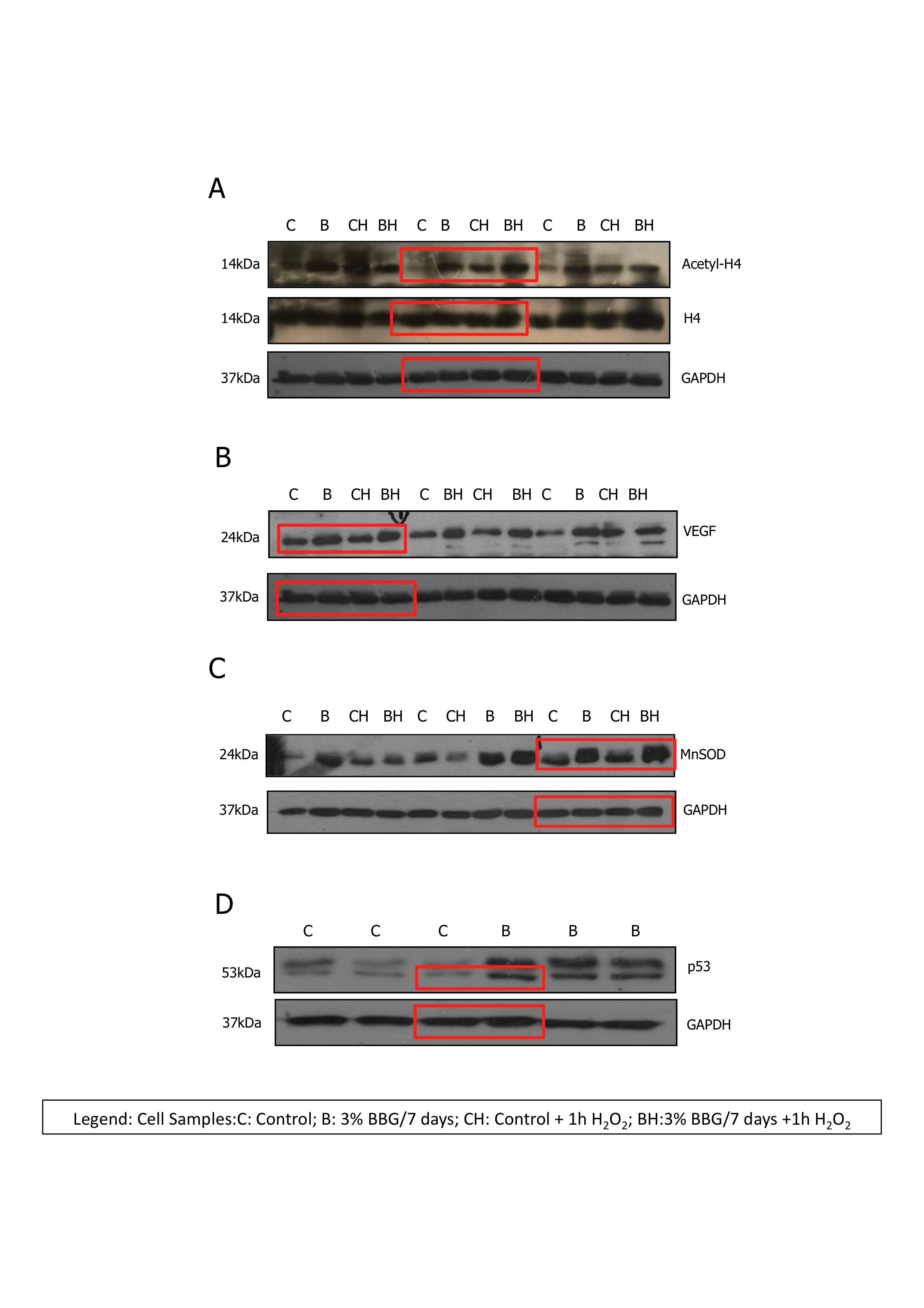


Supplemental Figure 2

**Supplementary Figure 2:** Western blot assay of HUVECs. Panel A, B, C, D show the full-length Western blots corresponding to cropped blots in the main text.
